# Comparación del espacio de la vía aérea faríngea en radiografías laterales de cabeza de individuos de clase I y II esquelética

**DOI:** 10.21142/2523-2754-1004-2022-128

**Published:** 2023-12-26

**Authors:** Santiago Razo Huillca

**Affiliations:** 1 Carrera de Estomatología, Universidad Científica de Sur. Lima, Perú. santiagorazoh@gmail.com Universidad Científica del Sur Carrera de Estomatología Universidad Científica de Sur Lima Peru santiagorazoh@gmail.com

**Keywords:** vías aéreas, cefalometría, maloclusión, relación esquelética, airways, cephalometry, malocclusion, skeletal relation

## Abstract

**Objetivo:**

Comparar el espacio de la vía aérea faríngea (nasofaringe y orofaringe) a través del análisis de radiografías laterales en individuos de clase II esquelética comparado con un grupo control de clase I.

**Materiales y método:**

Este estudio fue de tipo observacional, descriptivo, transversal y prospectivo. Su muestra estuvo conformada por 60 radiografías laterales de cabeza distribuidas en 30 radiografías clase I (ANB 2° ± 2° y con maloclusión de clase I) y 30 radiografías clase II esquelética (ANB > 5° y con maloclusión de clase II-1). En ellas se midió el espacio en mm de las vías aéreas de la orofaringe y nasofaringe a través del método de McNamara en radiografías laterales de cabeza.

**Resultados:**

El promedio de la orofaringe en clase I fue de 11,71 mm ± 3,18 mm. En el grupo de clase II fue de 10,73 mm ± 2,36 mm. No se encontraron diferencias significativas (p = 0,18). El promedio de la nasofaringe en clase I fue de 18,45 mm± 4,11 mm. En clase II fue de 19,10 mm ± 3,89 mm, tampoco se encontraron diferencias significativas (p = 0.53).

**Conclusión:**

El espacio en mm de la nasofaringe presenta valores similares en milímetros en sujetos con maloclusión de clase I y clase II. No existe diferencia en el espacio en mm de la orofaringe entre sujetos con maloclusión esquelética de clase I y clase II.

## Abstract (en)

**Objective:**

To compare the space of the pharyngeal airway (nasopharynx and oropharynx) through lateral X-ray analysis in skeletal class II individuals with a control group composed of skeletal class I individuals.

**Materials and methods:**

This study was of the observational, descriptive, transversal, and prospective type. The sample was made up by 60 lateral head radiographs distributed between 30 class I (ANB 2°±2° and class I malocclusion) and 30 skeletal class II radiographs (ANB>5° and malocclusion class II-1). Measurements of the airway space in the oropharynx and nasopharynx were taken in mm through the McNamara method on lateral head radiographs.

**Results:**

The average space found in the oropharynx in class I was 11.71mm ± 3.18mm. In the class II group, it was 10.73mm ± 2.36mm. No significant differences were found (p=0.18). The average space found in the nasopharynx in the class I group was 18.45mm ± 4.11mm. In the class II group, it was 19.10mm ± 3.89mm. There were no significant differences found (p=0.53).

**Conclusion:**

The airway space in mm. of the nasopharynx presents similar values in millimeters in subjects with Class I and Class II skeletal malocclusion. There is no difference in the airway spaces of the oropharynx in subjects with Class I and Class II Malocclusions.

## INTRODUCCIÓN

La cefalometría, como instrumento de diagnóstico, fue descrita durante el siglo pasado (Broadbent, 1931), en este panorama fueron dados los reportes iniciales con referencia a un uso clínico ^1^. También en los años 50, fueron detectados y acotados fracasos, problemas y recidivas presentes en tratamientos aplicados en ortodoncia, que tenían como punto de partida la ausencia de un análisis cefalométrico íntegro de las distribuciones óseas y faciales ^2^. Entre los análisis cefalométricos se ubican el de Bjork, el de Steiner, el de Downs y secundarios de diagnóstico, como el de Wits y el de Tweed ^3^. A diferencia de todos los anteriores, el análisis cefalométrico de McNamara se sustenta fundamentalmente sobre mediciones lineales, al mismo tiempo que se basa en la relación entre la geometría y el ámbito de características faciales de otros análisis de investigaciones anteriores ^4^. 

La cefalometría está incluida dentro de un grupo de exámenes auxiliares, los cuales tienen que ser incluidos con el fin de lograr un diagnóstico definitivo en ortodoncia, así como en diversas áreas de la estomatología ^5^. Se obtiene a través de la toma de la radiografía lateral de la cabeza del paciente, con el propósito de analizar y comparar los resultados con las normas establecidas ^6^. Es conocida la existencia de diversas clases de maloclusiones que pueden ser diagnosticadas en los pacientes, pero hay estudios que relacionan este tipo de patologías con las medidas de las distancias de la orofaringe y la nasofaringe en radiografías cefalométricas, que demuestran una relación entre estas alteraciones y las variaciones de la norma ^7^; pero, al ser estudios en ciertas poblaciones, existe variación con respecto a otras razas, debido a múltiples variables en las estructuras blandas y óseas de las personas. Además, estudios comparativos entre grupos claramente diferentes son muy escasos ^8^^-^^12^. Por lo tanto, el objetivo del presente trabajo de investigación fue evaluar el espacio en mm de la vía aérea faríngea en radiografías laterales de individuos con relación esquelética de clase I y II. Se planteó como hipótesis nula que las dimensiones faríngeas, evaluadas a través del método de McNamara de pacientes tipo clase II contrastándolas con la clase I esquelética en radiografías laterales de cabeza, son similares.

## MATERIALES Y MÉTODOS

Esta investigación fue de tipo observacional, descriptiva, transversal y prospectiva. El estudio fue aprobado por el comité de ética de la Universidad Científica del Sur. La población de estudio estuvo conformada por 60 radiografías laterales de cabeza de pacientes que acudieron a una consulta privada durante los meses de enero a diciembre de 2017 por razones de diagnóstico en ortodoncia o cirugía oral. La muestra fue determinada mediante una fórmula de tamaño muestral para comparar dos promedios (el promedio de la orofaringe en individuos de clase I y II) mediante el programa Fisterra, y se consignaron los siguientes datos: Nivel de confianza = 95%; Poder de la prueba = 80%; Precisión= 0,4 mm; Varianza del grupo control = 1,9; siendo el “n” requerido de 30 individuos.

**Figura 1:**
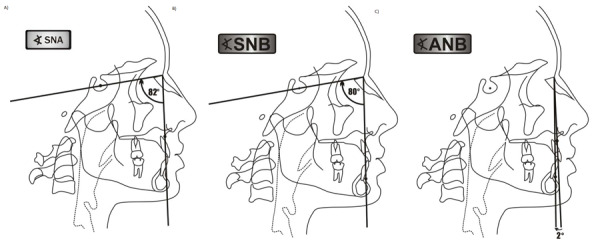
Determinación y valor normal del ángulo SNA, SNB y ANB.

Se incluyó lo siguiente: i) radiografías de pacientes de clase I esquelética (ANB 2°±2° y maloclusión de clase I según Angle); ii) radiografías de pacientes con clase II esquelética (ANB >5° y maloclusión clase II-1 según Angle); iii) Radiografías nítidamente visibles; iv) Radiografías laterales obtenidas en la consulta privada tomadas con el mismo ortopantomógrafo; v) Radiografías laterales de pacientes de ambos sexos; vi) Radiografías laterales de pacientes de 14 a 40 años (Fig. 1).

Se excluyó lo siguiente: i) Radiografías laterales distorsionadas; ii) Radiografías laterales de pacientes con aparato ortodóntico; y iii) Radiografías laterales de pacientes con asimetría severa, quistes o tumores.

El método fue la observación de tipo estructurada. Se presentó una carta para solicitar la autorización del centro privado para realizar el estudio en radiografías cefalométricas de aquellos pacientes evaluados en su consulta privada. Se recolectaron radiografías cefalométricas, las cuales presentaban el nivel del criterio establecido y se realizó con la ayuda del archivo digital de la consulta privada. Se desarrolló una prueba piloto para determinar el tamaño de muestra final para la investigación, para capacitar en la medición de las dimensiones de la orofaringe y nasofaringe, y para calibrar al investigador (kappa intraoperador e interoperador superiores a 0,7).

La interpretación radiográfica fue hecha por el investigador previamente capacitado y los resultados fueron consignados en un formato que incluyó el registro de la edad, el sexo y el tipo de maloclusión esqueletal. Cada radiografía fue medida de forma impresa y con calibración 1-1, con ayuda del programa Autocad (2017). Se procedió a medir el ángulo ANB de acuerdo con la clasificación de Steiner; así mismo, se consideró clase I cuando estuvo el valor en ANB de 2°±2° y con una maloclusión según Angle de clase I (overjet 2-3 mm, overbite 20%, y relaciones molar y canina de clase I). Luego, se conformó el grupo de clase II que presentaban el ANB > 5° y tenían la presencia de una maloclusión de clase II-1 de Angle (overjet > 3 mm, overbite adecuado, marcada inclinación de incisivos y relación molar y canina también clase II).

**Figura 2:**
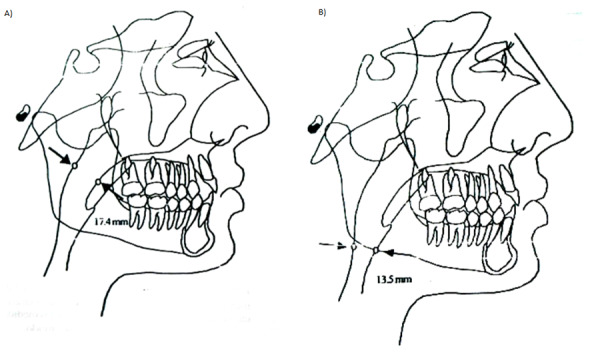
Ubicación y determinación de la medida de la nasofaringe y orofaringe

Fue definida la distancia de la nasofaringe como aquella presente dentro del contorno posterior, perteneciente al paladar blando y con la referencia más cercana encima de la pared ubicada en la parte posterior de la faringe. Se definió esta distancia de la orofaringe como la que existe entre la inserción en el contorno posterior de la lengua, el borde inferior mandibular y el punto más posterior en la pared posterior en la faringe (Fig. 2).

El análisis estadístico fue realizado a través del programa SPSS versión 20 y comenzó con el análisis univariado, que consistió en describir la estadística descriptiva (media, desviación estándar, mínimo, máximo, rango y varianza) de las variables dimensiones de la orofaringe y la nasofaringe, según relación esquelética. Posteriormente, se realizó el análisis bivariado por medio de la aplicación de la prueba T de Student para grupos independientes. Se trabajó con un parámetro establecido de significancia de 0,05.

## RESULTADOS

En la tabla se presentan la distribución por sexo y el promedio de edad en ambos grupos. En la clase I se presentaron 17 pacientes de sexo femenino y 13 de sexo masculino, con promedio de edad de 14,7 años. En clase II, se registraron 22 pacientes de sexo femenino y 8 de sexo masculino, con un promedio de edad de 15,2 años.

**Tabla 1:** Distribución de la edad y el sexo en cada grupo de estudio

Relación esquelética	n	Sexo		Total	
		Varón	Mujer	Edad media	D. E.
Clase I	30	13	17	14,7	2,42
Clase II	30	8	22	15,2	3,68
P valor				0,53	

*Prueba de T de Student

En la Tabla 2 se observa la evaluación del ángulo ANB de acuerdo con la relación esquelética. La clase I presentó un valor promedio de ANB de 3,61° ± 2,27° y la clase II, un ANB de 7,60° ± 1,90°. Claramente, se distingue una amplia y significativa diferencia al comparar ambos grupos: p < 0,001 mediante la prueba T de Student.

**Tabla 2:** Evaluación del ángulo ANB de acuerdo con la relación esquelética

Relación esquelética	ANB		
	n	Media	D. E.
Clase I	30	3,61	2,27
Clase II	30	7,60	1,90
p < 0,001			

*Prueba de T de Student

En la Tabla 3, se evalúan la orofaringe y la nasofaringe de acuerdo con la relación esquelética. Se observó a 30 sujetos evaluados en clase I con un promedio de orofaringe de 11,71 mm ± 3,18 mm. La clase II presentó un promedio de 10,73 mm ± 2,36 mm. Cuando se aplicó la prueba de T de Student se encontró un valor de p = 0,180 que demuestra que no existe diferencia significativa entre ambos grupos. Cuando se evaluó la nasofaringe, se observó para la clase I un promedio de 18,45 mm ± 4,11 mm y para la clase II, de 19,10 mm ± 3,89 mm (p = 0,532).

**Tabla 3:** Evaluación de la orofaringe y la nasofaringe de acuerdo con la relación esquelética

Relación esquelética	n	Orofaringe				Nasofaringe			
		Media	D. E.	Mín.	Máx.	Media	D. E.	Mín.	Máx.
Clase I	30	11,71	3,18	6,00	21,00	18,45	4,11	10,00	26,00
Clase II	30	10,73	2,36	7,00	16,00	19,10	3,89	12,00	26,00
P		0,180				0,530			

*Prueba de T de Student

## DISCUSIÓN

Un grupo importante de pacientes en Latinoamérica que acuden a consultas privadas son pacientes de relación esquelética de clase II en proporción ligeramente mayor a los clase I y son pocos los pacientes que presentan una clase III definida, motivo por el cual se decidió comparar en este estudio a pacientes con clase II, en contraposición con pacientes de clase I ^(1-5, 9, 11)^. La evaluación de la orofaringe y la nasofaringe, en relación con el tipo de afección esquelética en los maxilares, ha sido motivo de estudios científicos que intentan demostrar la relación de esta con el aumento o disminución de las longitudes determinadas a través de radiografías cefalométricas. Este estudio no fue la excepción, pues tuvo como propósito fundamental conocer si existen diferencias significativas en pacientes de dos grupos esqueléticamente diferentes, tanto en la clase I, en donde la maxila y la mandíbula se encuentran en una misma relación sagital, en comparación con la clase II, en donde los pacientes presentan generalmente una retrusión mandibular y, en otra proporción, protrusión maxilar.

Los resultados que se esperaba obtener en el estudio se englobaban en encontrar una diferencia significativa por una menor longitud del espacio de la orofaringe presente en pacientes con clase II esqueletal, debido a la frecuente retrusión mandibular que estos presentan, o incluso una mayor longitud de la nasofaringe debido a la protrusión maxilar que también pueden presentar; sin embargo, ambas condiciones no se presentaron en esta investigación, y se obtuvieron resultados semejantes al comparar ambas clases esqueléticas ^12^^-^^17^.

En este estudio hubo una semejanza en las distribuciones de todas las variables. Con relación al sexo, no hubo diferencia significativa y lo mismo sucedió con la edad de los pacientes; por lo tanto, se llegó a la conclusión de que ambos grupos eran adecuadamente comparables (9-12). Cuando se evaluó el ángulo ANB como factor de diferenciación de la relación esquelética, se encontró una evidente diferencia. Así mismo, el tamaño de muestra empleado de 30 pacientes por grupo no constituye un tamaño pequeño, pues se han presentado distintos estudios con un tamaño muy similar y que se encuentran disponibles en la literatura científica ^9^^-^^12^.

Lo que se esperaba encontrar en este trabajo era que aquellos pacientes con clase II esquelética tienen una menor longitud en la dimensión de la orofaringe de manera significativa y clínica de por lo menos unos 2 o 3 mm con respecto a la muestra de pacientes de clase I ^13^^-^^16^. Sin embargo, no se encontró una diferencia significativa, lo cual nos plantea la idea de que, por más que la mandíbula esté en retrusión, no se evidencia claramente una disminución en las dimensiones de la orofaringe en la muestra de los pacientes de este trabajo de investigación. Con respecto a las dimensiones de la nasofaringe, también se esperaba que los pacientes con clase II presenten un aumento significativo de las dimensiones de la nasofaringe producto de la protrusión maxilar presente en pacientes con clase II; sin embargo, esto no ocurrió, tal como pudimos observarlo en el estudio de Alcazar *et al*. ^17^^-^^20^, en el cual tampoco encontraron un incremento de las dimensiones estudiadas en la nasofaringe en pacientes de clase II. Probablemente y con base en la teoría sobre el tema se pueda encontrar diferencia para las dimensiones analizadas en la nasofaringe y la orofaringe en pacientes cuya presencia de clase II sea muy severa, es decir ángulos ANB superiores a 8° (casos quirúrgicos) ^21^^-^^23^, pero en este estudio no hemos abarcado este tipo de pacientes. Por el contrario, se recomendaría que se prosiga con la investigación acerca de este tema abarcando pacientes de clase II y clasificándolos de acuerdo con la severidad de la maloclusión.

Finalmente, el estudio concluye que no existe diferencia significativa entre el grupo diagnosticado de relación esquelética clase I y el grupo diagnosticado de relación esquelética de clase II, lo que clínicamente quiere decir que ambos grupos sometidos a una evaluación lineal poseen las similares características ^22^^,^^23^, no obstante, no se puede afirmar que volumétricamente esto se presente de igual manera; por lo tanto, más estudios en volumetría como en estereolitografía que puedan medir las dimensiones reales de este volumen faríngeo deben ser realizados en posteriores trabajos de investigación. Así mismo, pueden compararlo con la clase III esquelética. Finalmente, este estudio acepta la hipótesis nula, que indica que hay similitud en la vía aérea faríngea de los grupos comparados. 

## CONCLUSIONES

La nasofaringe presenta valores similares en milímetros en sujetos con maloclusión clase I y clase II.

No existe diferencia en el tamaño de la orofaringe entre sujetos con maloclusión clase I y clase II.

## Financiación:

Esta investigación fue autofinanciada
